# Prevalence of Chlamydia infection among women visiting a gynaecology outpatient department: evaluation of an in-house PCR assay for detection of *Chlamydia trachomatis*

**DOI:** 10.1186/1476-0711-9-24

**Published:** 2010-09-08

**Authors:** Achchhe L Patel, Divya Sachdev, Poonam Nagpal, Uma Chaudhry, Subash C Sonkar, Suman L Mendiratta, Daman Saluja

**Affiliations:** 1 Dr. B. R. Ambedkar Center for Biomedical Research, University of Delhi, Delhi-110007, India; 2 Department of Gynaecology, Hindu Rao Hospital, Malka Ganj, Delhi-110007, India

## Abstract

**Background:**

Screening women for *Chlamydia trachomatis *infection in developing countries is highly desirable because of asymptomatic infection. The existing diagnostic methods in developing countries are not effective and their sensitivity fall below 45.0% which leads to further spread of infection. There is an urgent need for improved and cost effective diagnostic tests that will reduce the burden of sexually transmitted infections in the developing world.

**Methods:**

Prevalence of *C. trachomatis *infection among women visiting gynaecology department of Hindu Rao hospital in Delhi, India was determined using Roche Amplicor Multi Well Plate kit (MWP) as well as using in-house PCR assay. We used 593 endocervical swabs for clinical evaluation of the in-house developed assay against Direct Fluorescence Assay (DFA; Group I n = 274) and Roche Amplicor MWP kit (Group II, n = 319 samples) and determined the sensitivity, specificity, positive predictive value (PPV), negative predictive value (NPV) of the in-house developed assay.

**Results:**

We detected 23.0% positive cases and there was a higher representation of women aged 18-33 in this group. An in-house PCR assay was developed and evaluated by targeting unique sequence within the *gyrA *gene of *C. trachomatis*. Specificity of the reaction was confirmed by using genomic DNA of human and other STI related microorganisms as template. Assay is highly sensitive and can detect as low as 10 fg of *C. trachomatis *DNA. The resolved sensitivity of in-house PCR was 94.5% compared with 88.0% of DFA assay. The high specificity (98.4%) and sensitivity (97.1%) of the in-house assay against Roche kit and availability of test results within 3 hours allowed for immediate treatment and reduced the risk of potential onward transmission.

**Conclusions:**

The in-house PCR method is cost effective (~ 20.0% of Roche assay) and hence could be a better alternative for routine diagnosis of genital infection by *C. trachomatis *to facilitate improved screening and treatment management.

## Background

Genital infection due to *Chlamydia trachomatis *is one of the most common sexually transmitted infections. Worldwide, an estimated 92 million new cases of *C. trachomatis *infection occur each year. More than two-thirds of these cases occur in the developing world, where diagnostic and treatment services are almost absent [1]. Asymptomatic (nearly 80.0% of women and 40.0% of men) [1] and untreated genital infections have serious ramifications for the reproductive health of women as it may evolve into complications such as ectopic pregnancy, pelvic inflammatory disease, salpingitis with tubal scarring and infertility in female patients [1-3]. In infected men, arthritis and epididymitis may result in urethral obstruction and decreased fertility. Chlamydial genital tract infection is an important risk factor for *human papillomavirus *induced cervical neoplasia as well as human immunodeficiency virus (HIV) transmission [4-6]. Undiagnosed and untreated chlamydial infections are thus not only creating major health problems and consequences for individuals but also result in major epidemiological, social and economical problems. The developing countries have a high incidence of new chlamydial infection, however, with the exception of sporadic testing, screening for *Chlamydia *is rare. Using various diagnostic tests with different performance characteristics, the prevalence of chlamydial infection among women in developing countries specifically sex workers varies from 8.5% to 37.0% [7-10]. The prevalence among female sex workers ranged from 27.0-36.0% in Philippines [11,12] while it is 24.0% in Indonesia [7].

The prevalence of sexually transmitted diseases could be as high as 17.6% among females from tribal population [13] to varying degree in metropolitan cities in India [14]. The incidence of chlamydial infection in female sex workers in Surat was estimated to be 8.5% using PACE2 test (non-amplified DNA probe assays for *C. trachomatis *and *N. gonorrhoeae*; Gen-probe San Diego, USA) while in Ahmedabad it was almost double [8]. Although national screening programmes are in place in developed countries, such programmes are non-existent in most of the developing countries even among high risk population such as sex workers. Consequently for symptomatic patients WHO recommends a syndromic approach to case management [15] but unnecessary treatment is the major disadvantage of syndromic management. The major limitation for screening programmes is the lack of simple and cost effective diagnostic tests. Intracellular localization of the pathogen creates an additional challenge for routine diagnosis. Diagnosis of chlamydial infection is even more difficult in asymptomatic and in chronic or persistent infections where the pathogen load would be low. The large pools of asymptomatic infected people are not only at the risk of developing serious long-term sequelae but would also transmit the infection. Urdea *et al.*, speculated that approximately 3 million victims suffering from Disability Adjusted Life Years (DALYs) can be saved, more than 12 million incidence of gonorrhoea and *Chlamydia *infections can be averted, about 161,000 HIV infections can be prevented among female commercial sex workers in sub-Saharan Africa, China and Southeast Asia with a diagnostic method that requires minimal laboratory infrastructure but has 85.0% sensitivity and 90.0% specificity for both gonorrhoea and *Chlamydia *[16]. A test that requires no laboratory infrastructure could save ~4 million DALYs, avert >16.5 million incidence of gonorrhoea and *Chlamydia *infections and prevent >212,000 HIV infections [16].

Nucleic acid amplification test (NAATs) are the tests of choice for the diagnosis of *C. trachomatis *genital infections because of their high sensitivity, specificity and suitability for various types of sample, including vulvovaginal swabs and first void urine (FVU), [3,14,17]. NAAT has also facilitated the use of less invasive procedures for detection of asymptomatic *C. trachomatis *infection in female patients. Several commercial NAATs are available and they make use of different technologies: conventional PCR; quantitative PCR (Roche Diagnostics, Abbott IL, USA); strand displacement amplification (Becton Dickinson, NJ, USA); transcription-mediated amplification (Gen Probe) and nucleic acid sequence-based amplification (Bio Merieux, Nancy L'Etoile, France). The high cost of these kits and lack of appropriate infrastructure are the major deterrents for using these kits for large screening programmes to be conducted in developing countries including India. The principal detection methods of *C. trachomatis *infections in India still remain to be the culture method and Direct Fluorescent Assay (DFA). In addition, most of the resource limited clinics continue to practice syndromic management. Therefore there is a paucity of information regarding epidemiology of STD including *C. trachomatis *infections in India. However, considering the high prevalence of infection in India there is an urgent need to design tests that are simple, inexpensive and can be used to improve diagnosis as well as specificity of the syndromic management [18-21].

In the present, study we have designed and established a simple PCR based assay for diagnosis of *C. trachomatis *using primers against genomic sequences. The evaluation of the test for its specificity, sensitivity, positive and negative predictive values against currently used diagnostic methods suggest that in-house PCR assay is highly comparable in its performance to that of commercial kits and is considerably less expensive. Availability of the test result within few hours could allow treatment at the initial visits and helps in preventing further transmission of the disease.

## Methods

### Enrolment of Patients

Total 593 out of the 2800 patients (median age 29 years and aged between 18-60 years), visiting gynaecology department, were enrolled for the study. Enrolment of patient was determined on the basis of vaginal discharge and other STD related symptoms. All participants were informed and oral consent of patients was taken.

### Specimen collection

A thorough vulval examination was done for lesions and vaginal/cervical discharge. Perspeculum examination was carried out for wart, erosions and abnormal growth by the clinicians. After cleaning the exocervix with cotton swab (Hi Media, Mumbai, India), two endocervical swabs were taken from each patient. In order to avoid swab sample variation that can occur when multiple swabs are taken, the clinical samples were divided into two groups (I & II). In the first group of 274 samples, sterile cotton swab was used to collect each specimen in 1 ml transport medium [22]. The specimen from second swab was smeared on a clean glass slide, air-dried for DFA and Fluorescence *in-situ *hybridization assay (FISH). For the second group of 319 samples, two endocervical swab specimens were obtained. The first swab was placed in a vial containing AMPLICOR Specimen Transport Medium of Roche and the second swab was placed in 1 ml transport medium. All samples were kept on ice and tested within 24 hours or stored at -80°C for subsequent use.

### Primer designing

Sequences of four genes of *C. trachomatis *were obtained from Gene Bank. The selected sequences were Glucosamine-Fructose-6-phosphate Aminotransferase (*gfa*) (CT816, Entrez Gene ID: 884625), hypothetical protein (CT163, Entrez Gene ID: 884039), PhospholipaseD Endonuclease (*plde*) Superfamily (CT157, Entrez Gene ID: 884104) and g*yrA *gene (CT189, Entrez Gene ID: 884941), producing an amplicon of size 145-bp, 418-bp, 368-bp and 463-bp respectively. Short stretches of about 25-30 nucleotides were aligned using the BLAST program http://blast.ncbi.nlm.nih.gov/ from NCBI to assess potential cross-reactivity with other organisms. Sequences unique to *C. trachomatis *were selected for primer designing using Gene Runner 3.05. The selected primers of *gyrA *(CT189) are as follows: Forward primers C2- 5' TGATGCTAGGGACGGATTAAAACC 3', Tm 63.7°C, Reverse primer C5- 5' TTCCCCTAAATTATGCGGTGGAA 3', Tm 64°C. (Indian patent number 239908; US, UK, EU patent pending).

### PCR Amplification

For PCR assay, specimens were processed by lysis method as described previously [22]. Supernatant (5 μl) of processed sample or crude lysate was used for PCR in a reaction volume of 25 μl containing 1× Taq DNA polymerase buffer (50 mM KCl, 10 mM Tris-HCl pH 8.3, 1.5 mM MgCl_2_), 200 μM each of the four dNTPs (New England Biolabs Inc, Beverly, MA, USA), 10 pmoles each of forward and reverse primers, 0.7 U of Taq DNA polymerase (Bangalore Genei India Pvt. Ltd., Bangalore, India). Purified genomic DNA of *C. trachomatis *(kindly provided by Lynn Olinger, Francis I. Proctor Foundation, University of California, San Francisco) was used as a positive control for each set of assays. Amplification was performed using thermal cycler (I cycler, Bio-Rad, Richmond, USA) for 35 cycles; 95°C for 5 min for initial denaturation, followed by 35 cycles of 95°C for 30 sec, 60°C for 30 sec, 72°C for 1 min, final extension at 72°C for 5 min. The amplicons were analyzed on 1.5% agarose gel by electrophoresis. The amplicons from ten percent of positive samples were eluted using a DNA isolation kit (Biological Industries Ltd., Kibbutzbeit Haemek, Israel) according to the manufacturer's instructions and sequenced using PCR primers (forward primer) with a Taq-Dye terminator cycle sequencing kit on 377A autosequencer (Applied Biosystems, California, USA) at TCGA (IGIB) India. DNA sequence of the amplified product was compared to known *gyrA *nucleotide sequences (Jan, 2009) in the GenBank databases using BLAST program to determine the percent identity.

### Roche AMPLICOR MWP *Chlamydia trachomatis *Detection Assay

Three hundred and nineteen endocervical specimens of group II were tested by Roche AMPLCOR CT detection kit (Roche Diagnostic Systems) according to the manufacturer's instructions. For endocervical specimens, 1 ml of specimen lysis buffer was added to endocervical samples, mixed thoroughly by vortexing and were incubated for 10 min at room temperature. After overnight storage at 4°C, 50 μl of the clinical sample was added to each PCR tube containing 50 μl of PCR master mix. The PCR master mix contained primers for internal control as well. The assay was developed as per instructions given by manufacturers. Samples for which the two methods described above showed discrepancy, genomic DNA was isolated from the aliquots of frozen specimens. Samples were centrifuged at 15,000 × g for 30 min. Pellet was resuspended in 500 μl of lysis buffer (Tris EDTA 50 mM pH 8.0, Proteinase K 400 μg/ml) and incubated at 55°C for two hours and then boiled at 100°C for 10 min with 1 mM DTT. Thereafter DNA was extracted with phenol:chloroform and centrifuged at 12,000 × g for 10 min, DNA was precipitated with isopropanol in the presence of 0.3 M sodium acetate and incubated overnight at -20°C. Pellet was collected by centrifugation at 12,000 × g for 10 min, washed with 70% ethanol, air-dried and dissolved in PCR grade water [22].

### Evaluation of specificity and sensitivity

To evaluate the specificity of the primer pair, DNA extracted from positive controls, pathogens causing STI and those representing general microflora of cervix were used as templates in PCR reaction (Table 1). DNA was extracted from these microorganisms as described above. Ten human genomic DNA samples were also used to evaluate the specificity of primer pair. To determine the sensitivity of primer pair, purified genomic DNA from *C. trachomatis *at 100 pg to 1 fg concentrations following serial dilutions and various dilutions of *C. trachomatis *positive clinical samples were used as the templates for PCR amplification. All assays were repeated at least five times. For clinical samples, repeat assays were performed for randomly selected samples.

**Table 1:** List of various organisms and their source; used for evaluation of specificity of the C2/C5 primers.

Organism/Strain/Isolate	Number of Specimens tested	Source(s)
*C. trachomatis serovar *L2	1	Dr. Peter Braun, Max Plank Institute for Infection Biology, Berlin, Germany

*C. trachomatis Serovar *A & D	12 isolates from STD cases	Dr. Sudha Salhan, Department of Obstetrics and Gynaecology, Vardhman Mahavir Medical College and Safdarjang Hospital, New Delhi, India.

*Mycoplasma spp*.	8	Department of Microbiology AIIMS, New Delhi, India.
*Chlamydia pneumoniae*	5	
*Candida spp*.	10	
*Ureaplasma*	11	
*Pseudomonas aeruginosa*	2	
*Klebsiella pneumoniae*	2	
*Acinetobacter baumannii*	2	
*Staphylococcus aureus*	2	
Herpes simplex virus 1(HSV-1) and Herpes simplex virus 2 (HSV-2)	2	
Cytomegalovirus	2	
BK virus	2	

*Trichomonas spp*.	8	Department of Microbiology AIIMS, New Delhi, India & Department of Poultry Science, University of Georgia, Athens, USA.

*Neisseria gonorrhoeae*	2	

*Neisseria meningitidis *(genital isolates)	3	Prof. J. W. Tapsall, WHO Collaborating Centre for STD and HIV, Department of Microbiology, The Prince of Wales Hospital, Randwick, New South Wales, Australia.
*Neisseria lactamica *94D4	1	
*Neisseria sicca *94C1	1	
*Neisseria subflava *86G7	10	

### Direct Fluorescence Assay

For DFA (MicroTrak, Co Wicklow, Ireland), specimens were centrifuged at 3000 × g for 10 min and pellets were air dried, fixed by incubation in methanol and stained with a fluorescein isothiocyanate-conjugated anti-MOMP monoclonal antibody. The slides were examined for typical apple-green fluorescent elementary bodies (EBs) at 1000× magnification. The presence of more than ten fluorescent EBs was scored as a positive case.

### Fluorescence In-situ Hybridization

FISH assay was performed as described previously [23].

### Definition of a positive sample

All 274 clinical specimens in group I were tested by DFA and in-house PCR. FISH assay was carried out on discrepant results as a confirmatory test. Samples were considered positive if they tested positive by any of the two methods. All 319 clinical specimens of Group II were tested by Roche Amplicor MWP kit and by the in-house PCR assay. Amplification of known *C. trachomatis *genes *ompA *(CT681; Entrez GeneID: 884473) and *plde *(CT157, Entrez Gene ID: 884104) were carried out on discrepant samples. Samples were considered positive if they tested positive by at least two PCR methods: Roche Amplicor MWP kit/in-house PCR/PCR amplification of *ompA *or *plde *gene. The prevalence of *C. trachomatis *infection was determined based on the total number of positive samples after discrepant analysis.

### DATA analysis

All statistical analysis was performed using GraphPad Prism version 5.03 software. Sensitivity, specificity, PPVs and NPVs were calculated with 95% confidence intervals to test the significance.

## Results and Discussion

**Figure 1:**
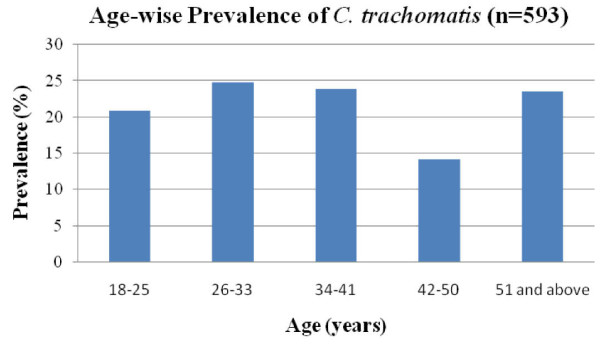
**Prevalence of *C. trachomatis *infections by age group; age range from 18-51 years and above**.

**Figure 2:**
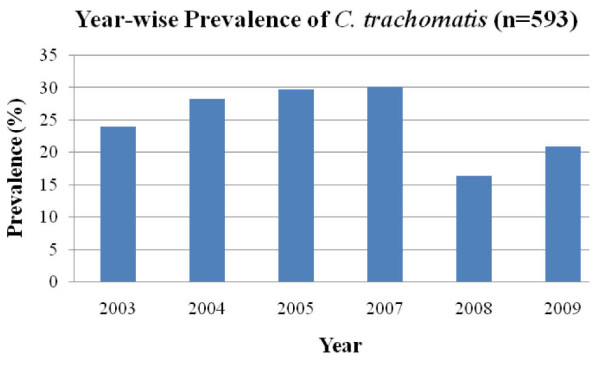
**Prevalence of *C. trachomatis *infections by year (2003-2009)**.

The cardinal sign for chlamydial infection according to syndromic case management guidelines is discharge from the cervix. Out of 2800 female patients who visited the gynaecology department for reproductive health problems and contraception, 593 patients were enrolled for this study. Almost all participants were symptomatic, including vaginal discharge (58.0%), lower abdominal pain (32.0%), infertility (4.3%) and other STD related symptoms. In our study overall prevalence of STD among females with STI related symptoms was 21.2% (593/2800). STI was more common among patients in the age group of 18-33 years (391/567). In an earlier study, STI prevalence was found to be 16.2% among patients attending STD clinics at a regional STD centre at New Delhi [24]. A relatively high prevalence (36.5%) of STI is reported in tribal population of central India [25]. In a study from Surat, 47.5% of female sex workers were reported to have STI [8], while in Calcutta 59.0% sex workers had STD [26]. In the present study, genital chlamydial infection, as detected by Roche Amplicor test, DFA and in-house PCR assay, was 25.2% among the symptomatic females enrolled. Although *C. trachomatis *prevalence in patients of different age groups was not significantly different (Figure 1), more than 92.0% (118/128) of the total patients positive for *C. trachomatis *were from reproductively active age group of 18-41 years. During the course of the study (2003-2009), the prevalence of patients infected with *C. trachomatis *ranged from 24.0% to 30.0% (Figure 2). Among female sex workers in Surat, India, the prevalence of genital chlamydia by PACE2 test was found to be 8.5%, while in Ahmedabad, India it was reported to be twice as much [8]. Among the tribal populations (patients/general population) from central India although STI was high (36.5%), only 4.0% chlamydial infection is reported [24] which is similar to that observed among patients in Azerbaijan 3.1% [27] and Bangladesh 3.4%, [28]. High chlamydial infection has also been reported in Manila 23.3%, Cebu, Philippines 37.0% and 14.0% in Nicaragua [21,29].

### Specificity and sensitivity of primers

**Figure 3:**
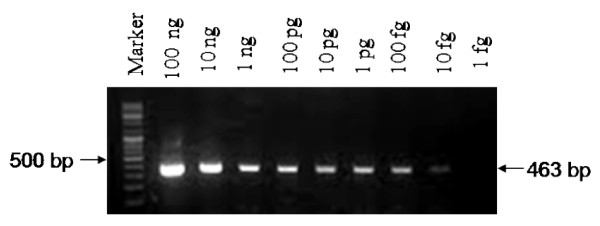
**Sensitivity of in-house primers using purified chlamydial genomic DNA**. PCR amplification of purified genomic DNA (100 ng to 1 fg) of *C. trachomatis *serovar D was carried out by using C2/C5 primers.

Using the BLAST program from NCBI, *gyrA *gene of *C. trachomatis *(Entrez GeneID: 884941) was selected for primer designing. Amplicons of desired size (463-bp) were obtained when purified genomic DNA of different serovars of *C. trachomatis *(A, D and L2) were used as templates for the in-house PCR, while no amplicon was detected when genomic DNA from humans as well as other STD causing and related microorganisms were tested as templates. The specificity was further confirmed by sequencing the amplicons obtained from 10.0% of positive clinical samples. The DNA sequence of the amplicons were aligned to the known sequences of serovars A, B, D, E, G, L2 and Sweden2 of *C. trachomatis *in the GenBank databases (Additional file 1). The primer pair was highly sensitive as an amplicon could be seen when as low as 10 fg of purified chlamydial genomic DNA (equivalent to 9 IFUs) was used as template (Figure 3). C2/C5 primers amplified the target sequence even when crude lysates of clinical samples were used in the PCR reaction.

NAAT based diagnosis has been observed to be significantly more sensitive than conventional methods such as culture and antigen based methods for diagnosis of *C. trachomatis *in clinical specimens. Several existing PCR assays including the Roche MWP test target the cryptic plasmid while the chromosomal targets most widely used are ompA and rRNA coding genes [20-35]. Plasmid based PCR detection assays are considered to be more sensitive compared to chromosomal gene based assay, as plasmid copy number is generally 7 to 10 per cell [36-38]. NAAT based diagnostic kits like COBAS AMPLICOR CT (Roche Diagnostic system) and Ligase chain reaction kit from Abbott (presently out of market) were shown to be highly sensitive (92.9 to 100%) and specific (99.0 to 100%) for *C. trachomatis *detection [3,39-42]. However, major obstacle in adopting these kits for routine diagnosis in clinical laboratories in India is their high cost (average cost of single assay is $50). It may also be emphasized that the commercially available diagnostic kits use plasmid-based amplification. A new variant of *C. trachomatis *(Sweden2), carrying a 377-bp deletion within the plasmid, was reported in Sweden [43]. This deletion includes the target sequences used in the commercial diagnostic assays of Roche and Abbott. We have also observed infection due to plasmid free variants as reported in other populations [44,45], which remains a challenge for detection (unpublished results).

Comparison of the performance of different PCR assays including plasmids, *ompA *and *rDNA *targets has suggested that in general plasmid primers are 10 to 1000 times more sensitive (0.1 fg for plasmid DNA) than the genomic DNA primers for *ompA *(0.1 pg to 10 pg genomic DNA) and *rDNA *(1 pg genomic DNA) [34,46-48]. Our assay targeting *gyrA *gene showed high sensitivity (10 fg to 0.1 pg genomic DNA) compared to other genomic targets.

### Clinical performance of in-house PCR and its comparison with DFA and Roche Amplicor MWP kit

To check the performance of in-house PCR assay, the samples were divided into groups as mentioned under methods. The median age of patients for group I (274 patients) was 29 years (range: 18 to 57 years). Out of 274 patient samples, 59 (21.5%) were positive and 191 samples (69.7%) were negative both by PCR assay using C2/C5 primer set as well as by DFA (Table 2). Out of the 28 discrepant samples, 15 samples were considered true positive, as 11 samples were PCR and FISH positive but DFA negative while 4 were DFA and FISH positive but PCR negative (Table 3). The remaining thirteen discrepant samples were considered true negative as they tested negative both by DFA and FISH assay. These 13 cases, which were PCR positive, could not be reconfirmed as follow-up specimens could not be collected because the patients had received treatment. Based on our results, it is evident that C2/C5 primer pair detected significant number of true positive samples (70 out of 74, p < 0.0001) as compared to the DFA method that detected 63 out of 74 as true positive samples.

**Table 2:** Comparison of in-house PCR with DFA and Roche amplicor MWP kit, results for *C. trachomatis *before and after discrepant analysis.

PCR results C2/C5					After resolution of discordant results	
**Group I**	**DFA**	**Positive**	**Negative**	**Total**	**Positive**	**Negative**

Positive		59	24	83	70(59+11)	13(24-11)
Negative		4	187	191	4	187
Total		63	211	274	74	200

Group II	Roche					

Positive		60	13	73	69(60+9)	4(13-9)
Negative		2	244	246	2	244
Total		62	257	319	71	248

**Table 3:** Resolution of discrepant results for *C. trachomatis *infection.

No. of discrepant samples					Conclusion
		**C2/C5**	**DFA**	**FISH**	

Group I (n = 28)	11	+	-	+	True positive
	4	-	+	+	True positive
	13	+	-	-	True negative

Group II (n = 15)			Roche	*ompA/plde*	

	9	+	-	+	True positive
	4	+	-	-	True negative
	2	-	+	+	True positive

A second test was applied for each sample. FISH was taken as the gold standard for group I and *ompA/plde *for group II.

Among the 319 samples enrolled in group II, 244 were negative while 60 samples were positive by both in-house PCR and commercial PCR assay (Table 2). To resolve the discrepancy, the genomic DNA of discrepant samples (as described under methods) was purified to remove PCR inhibitors if any, and tested for amplification of two housekeeping genes: *ompA *and *plde *as well as by in-house PCR and commercial PCR method. Out of 15 discrepant samples, 13 samples that were positive by in-house PCR but negative by commercial PCR, 9 samples tested positive for *ompA*/*plde *and hence were considered true positive. The remaining four in-house PCR positive samples, tested negative for *ompA *and *plde *and Roche PCR and were thus scored as true negatives. Two discrepant samples that were positive by Roche Amplicor MWP kit and negative by in-house PCR, tested positive for *ompA/plde *and were therefore considered true positive (Table 3). The response to therapy administered to the patients with discrepant results obtained from hospital records also supported our final results shown in Table 3.

Once again as for group I, C2/C5 primers detected a significant number of samples (69 out of 71, 97.0%) and its efficiency is comparable to that of commercial PCR assay which detected 98.0% of positive samples. However, the commercial PCR kit detected many samples only when DNA was purified from these samples. Based on our results, the prevalence of *C. trachomatis *infection was 25.5% among women visiting gynaecology outpatient clinic when DFA was selected as gold standard while it was found to be 21.6% when Roche Amplicor MWP kit was taken as gold standard. The overall prevalence of *C. trachomatis *was found to be 23.0% among symptomatic patients enrolled in the study.

### Sensitivity, specificity, PPV and NPV

The estimated sensitivity and specificity range of DFA is 61.0% to 92.0% and 99.0% to 100% respectively in different laboratory settings when compared to that of culture or non-culture methods [44]. This could be due to sample preparation and handling conditions in the clinical set up as well as the laboratory. To avoid such factors, the discrepant samples were analyzed with alternative methods of detection in order to determine their true status. The in-house PCR (C2/C5) primers demonstrated sensitivity of 93.6% which increased to 94.5% after resolution of the discrepant cases by a second method for group I (Table 4). Similarly, the specificity of PCR by C2/C5 primers increased from 88.6% to 93.6% after discrepant analysis by FISH. The positive predictive value (PPV) of in-house PCR increased from 71.0% to 84.3% while the negative predictive value (NPV) remained unchanged at 98.0% even after confirming the status of discrepant samples. All the discrepant samples (see Table 3) when retested by in-house PCR method gave 100% reproducible results. The estimates of specificity of in-house PCR might have improved further if the follow up samples from patients could be obtained. The overall sensitivity of DFA in the present study was 88.0% which is close to suggested value of 92.0% [44]. In general, the cut-off for DFA is established to get the best combination of sensitivity and specificity, as a result, sometimes one may miss out a positive sample by compromising sensitivity to achieve specificity. Thomas *et al. *[33] have reported that DFA kit has its limitation as a reference method for evaluating a new diagnostic kit or to check inter-laboratory variation. According to their report the diagnostic performance of DFA test is highly dependent on the number of chlamydial EBs that should be seen in order to score a sample as positive sample. Since about 30.0% of the clinical samples contain ≤ 10 EBs, they are scored as negative. Shattock *et al. *[32] observed similar limitation when they compared various detection methods for *C. trachomatis*. Thus a sample may be DFA negative as it contains ≤ 10 EBs but can score positive by PCR method. In our study we observed 28 samples out of 274 that were positive by C2/C5 primers but negative by DFA or FISH assay. These samples, which repeatedly were positive by our PCR, may have contained chlamydial genomic DNA, but unfortunately no follow up samples were available to confirm this as patients responded to treatment and in some cases they did not return to the clinic.

**Table 4:** Performance of in-house PCR assays based on expanded spectrum of positivity after confirmatory FISH assay and *ompA/plde *PCR.

In-house PCR Assay	Sensitivity		Specificity		PPV		NPV	
	**%**	**95% CI^a^**	**%**	**95% CI^a^**	**%**	**95% CI^a^**	**%**	**95% CI^a^**

Group I	94.5	86.7-98.5	93.6	89.1-96.5	84.3	74.7-91.4	98	94.7-99.4
Group II	97.1	90.2-99.7	98.4	95.9-99.6	94.5	86.6-98.5	99	97.1-99.9

^a ^CI, Confidence interval.

We also evaluated our in-house PCR method against commercially available and widely used Roche MWP kit. It is pertinent to mention that to avoid the swab sample variation that may occur when multiple swabs are taken (especially when infection load is low); two swabs were taken for each patient. We observed inhibition of amplification reaction when internal control provided by Roche MWP kit also did not amplify. Similar observation is reported previously [32,48]. However, when DNA from these samples was purified and retested by Roche MWP kit, they were found to be positive. Compared with Roche MWP PCR assay our in-house PCR by C2/C5 shows 96.7% sensitivity and 95.0% specificity. Subsequent to resolving discrepancy the sensitivity of PCR method increased to 97.1% and specificity increased to 98.4% and the PPV of in-house PCR increased from 82.1% to 94.5% while the NPV remained unchanged at 99.0% (Table 4).

## Conclusions

The major objective of this work was to develop an in-house PCR assay that is cost effective and has high sensitivity and specificity so that it can be used for diagnosis of *C. trachomatis *infection. Despite the high prevalence of STI in developing countries, the laboratory confirmation of infection is not carried out because of poor resource settings and due to lack of simple, cost effective diagnostic tools. The existing diagnostic methods in developing countries are not effective and their sensitivity fall below 45.0% which leads to further spread of infection [49]. The annual incidence of STIs in India is about 5.0% with 40 million new cases every year [50]. This prompted WHO to emphasise on syndromic approach for case management in developing countries including India. A number of authors suggest that syndromic management based on vaginal discharge syndrome either miss out a significant proportion of cases of genital chlamydia or lead to treatment even in the absence of infection of *C. trachomatis *[8,51,52]. In the present study we enrolled patients having symptoms and expecting treatment. In-spite of this selection bias, the chlamydial infection was observed to be high among females in Delhi as was evident by laboratory investigations. Quick and inexpensive diagnostic test developed in the present study can help in regular clinical and laboratory screening for *C. trachomatis*. Consistent high prevalence of chlamydial infection as well as the potential synergistic role of STI in HIV and HPV transmission [4-6] suggest that syndromic management along with periodic screening may prove to be more effective approach to achieve long term goals of STI and HIV control through sustained access to effective preventive and treatment services.

The principle goal of this study was to develop an in-house PCR method that is at least as sensitive and as specific as commercial method. An additional advantage of the in-house PCR method would be its low cost. Since our studies also suggest that there is a high prevalence of *C. trachomatis *(>23.0% in females visiting gynaecology department), there is a definite need to have a cost effective method for routine diagnosis in India. We consider that implementation of the specific and sensitive PCR assay, developed in the present study may allow clinical microbiology laboratories in developing countries to detect *C. trachomatis *rapidly, which would be of great consequences in disease management.

## Competing interests

The authors declare that they have no competing interests.

## Authors' contributions

ALP participated in designing the experiments, executing them, performing data analysis and writing the manuscript. DS and PN contributed in designing and performing the experiments. UC helped in designing of experiments and writing of the manuscript. SCS participated in performing the clinical evaluation of the in-house PCR. SM organized and supervised the collection of clinical specimens. DS (corresponding author) instigated the project, designed experimental settings, finalized data analysis and writing of the manuscript. All authors read and approved the final manuscript.

## Supplementary Material

Additional file 1 **ClustalW showing the homology between amplified sequence of DNA GyrA Subunit A in various serovars of *C. trachomatis *and clinical samples**. Alignment of the DNA sequences in the GenBank database of the *gyrA *amplicon for serovars A, B, D, E, G, L2 and Sweden2 of *C. trachomatis*. Click here for file
