# Supplementary material for: Prevalence of Chlamydia infection among women visiting a gynaecology outpatient department: evaluation of an in-house PCR assay for detection of Chlamydia trachomatis
Source: Ann Clin Microbiol Antimicrob. 2010 Sep 8;9:24. doi: 10.1186/1476-0711-9-24 (PMC2944303; doi:10.1186/1476-0711-9-24)
Supplement: Additional file 1 — ClustalW showing the homology between amplified sequence of DNA GyrA Subunit A in various serovars of C. trachomatis and clinical samples. Alignment of the DNA sequences in the GenBank database of the gyrA amplicon for serovars A, B, D, E, G, L2 and Sweden2 of C. trachomatis. [file 1476-0711-9-24-S1.DOC]

Additional File 1 : ClustalW showing the homology between amplified sequence of DNA GyrA Subunit A in various serovars of C. trachomatis and clinical samples.

Reference sequence used for designing primers for the in house PCR is : Chlamydia trachomatis D/UW-3/CX

Gene : *gyrA*

Product=" DNA Gyrase subunit A “

Locus_tag : CT_189

Entrez Gene Id: 884941

Clinical TGATGCTAGGGACGGATTAAAACCTTCTCAGCGACGTATTTTATACGCTATGAAACAATT 60

D-LC TGATGCTAGGGACGGATTAAAACCTTCTCAGCGACGTATTTTATACGCTATGAAACAATT 60

D-EC TGATGCTAGGGACGGATTAAAACCTTCTCAGCGACGTATTTTATACGCTATGAAACAATT 60

G/9301 TGATGCTAGGGACGGATTAAAACCTTCTCAGCGACGTATTTTATACGCTATGAAACAATT 60

G/11074 TGATGCTAGGGACGGATTAAAACCTTCTCAGCGACGTATTTTATACGCTATGAAACAATT 60

A/HAR-13 TGATGCTAGGGACGGATTAAAACCTTCTCAGCGACGTATTTTATACGCTATGAAACAATT 60

D/UW-3/CX TGATGCTAGGGACGGATTAAAACCTTCTCAGCGACGTATTTTATACGCTATGAAACAATT 60

B/Jali20/OT TGATGCTAGGGACGGATTAAAACCTTCTCAGCGACGTATTTTATACGCTATGAAACAATT 60

B/TZ1A828/OT TGATGCTAGGGACGGATTAAAACCTTCTCAGCGACGTATTTTATACGCTATGAAACAATT 60

L2b/UCH-1/proctitis TGATGCTAGGGACGGATTAAAACCTTCTCAGCGACGTATTTTATACGCTATGAAACAATT 60

E/11023 TGATGCTAGGGACGGATTAAAACCTTCTCAGCGACGTATTTTATACGCTATGAAACAATT 60

Sweden2 TGATGCTAGGGACGGATTAAAACCTTCTCAGCGACGTATTTTATACGCTATGAAACAATT 60

E/150 TGATGCTAGGGACGGATTAAAACCTTCTCAGCGACGTATTTTATACGCTATGAAACAATT 60

************************************************************

Clinical AAATCTGACTCCAGGAGTAAAGCACAGAAAATGCGCAAAAATTTGCGGTGATACTTCCGG 120

D-LC AAATCTGACTCCAGGAGTAAAGCACAGAAAATGCGCAAAAATTTGCGGTGATACTTCCGG 120

D-EC AAATCTGACTCCAGGAGTAAAGCACAGAAAATGCGCAAAAATTTGCGGTGATACTTCCGG 120

G/9301 AAATCTGACTCCAGGAGTAAAGCACAGAAAATGCGCAAAAATTTGCGGTGATACTTCCGG 120

G/11074 AAATCTGACTCCAGGAGTAAAGCACAGAAAATGCGCAAAAATTTGCGGTGATACTTCCGG 120

A/HAR-13 AAATCTGACTCCAGGAGTAAAGCACAGAAAATGCGCAAAAATTTGCGGTGATACTTCCGG 120

D/UW-3/CX AAATCTGACTCCAGGAGTAAAGCACAGAAAATGCGCAAAAATTTGCGGTGATACTTCCGG 120

B/Jali20/OT AAATCTGACTCCAGGAGTAAAGCACAGAAAATGCGCAAAAATTTGCGGTGATACTTCCGG 120

B/TZ1A828/OT AAATCTGACTCCAGGAGTAAAGCACAGAAAATGCGCAAAAATTTGCGGTGATACTTCCGG 120

L2b/UCH-1/proctitis AAATCTGACTCCAGGAGTAAAGCACAGAAAATGCGCAAAAATTTGCGGTGATACTTCCGG 120

E/11023 AAATCTGACTCCAGGAGTAAAGCACAGAAAATGCGCAAAAATTTGCGGTGATACTTCCGG 120

Sweden2 AAATCTGACTCCAGGAGTAAAGCACAGAAAATGCGCAAAAATTTGCGGTGATACTTCCGG 120

E/150 AAATCTGACTCCAGGAGTAAAGCACAGAAAATGCGCAAAAATTTGCGGTGATACTTCCGG 120

************************************************************

Clinical AGATTATCACCCTCATGGAGAAAGTGTCATTTATCCTACTTTAGTAAGGATGGCACAGGA 180

D-LC AGATTATCACCCTCATGGAGAAAGTGTCATTTATCCTACTTTAGTAAGGATGGCACAGGA 180

D-EC AGATTATCACCCTCATGGAGAAAGTGTCATTTATCCTACTTTAGTAAGGATGGCACAGGA 180

G/9301 AGATTATCACCCTCATGGAGAAAGTGTCATTTATCCTACTTTAGTAAGGATGGCACAGGA 180

G/11074 AGATTATCACCCTCATGGAGAAAGTGTCATTTATCCTACTTTAGTAAGGATGGCACAGGA 180

A/HAR-13 AGATTATCACCCTCATGGAGAAAGTGTCATTTATCCTACTTTAGTAAGGATGGCACAGGA 180

D/UW-3/CX AGATTATCACCCTCATGGAGAAAGTGTCATTTATCCTACTTTAGTAAGGATGGCACAGGA 180

B/Jali20/OT AGATTATCACCCTCATGGAGAAAGTGTCATTTATCCTACTTTAGTAAGGATGGCACAGGA 180

B/TZ1A828/OT AGATTATCACCCTCATGGAGAAAGTGTCATTTATCCTACTTTAGTAAGGATGGCACAGGA 180

L2b/UCH-1/proctitis AGATTATCACCCTCATGGAGAAAGTGTCATTTATCCTACTTTAGTAAGGATGGCACAGGA 180

E/11023 AGATTATCACCCTCATGGAGAAAGTGTCATTTATCCTACTTTAGTAAGGATGGCACAGGA 180

Sweden2 AGATTATCACCCTCATGGAGAAAGTGTCATTTATCCTACTTTAGTAAGGATGGCACAGGA 180

E/150 AGATTATCACCCTCATGGAGAAAGTGTCATTTATCCTACTTTAGTAAGGATGGCACAGGA 180

************************************************************

Clinical TTGGGCCATGCGATACCCTCTTGTTGATGGTCAAGGGAATTTTGGATCCATCGACGGGGA 240

D-LC TTGGGCCATGCGATACCCTCTTGTTGATGGTCAAGGGAATTTTGGATCCATCGACGGGGA 240

D-EC TTGGGCCATGCGATACCCTCTTGTTGATGGTCAAGGGAATTTTGGATCCATCGACGGGGA 240

G/9301 TTGGGCCATGCGATACCCTCTTGTTGATGGTCAAGGGAATTTTGGATCCATCGACGGGGA 240

G/11074 TTGGGCCATGCGATACCCTCTTGTTGATGGTCAAGGGAATTTTGGATCCATCGACGGGGA 240

A/HAR-13 TTGGGCCATGCGATACCCTCTTGTTGATGGTCAAGGGAATTTTGGATCCATCGACGGGGA 240

D/UW-3/CX TTGGGCCATGCGATACCCTCTTGTTGATGGTCAAGGGAATTTTGGATCCATCGACGGGGA 240

B/Jali20/OT TTGGGCCATGCGATACCCTCTTGTTGATGGTCAAGGGAATTTTGGATCCATCGACGGGGA 240

B/TZ1A828/OT TTGGGCCATGCGATACCCTCTTGTTGATGGTCAAGGGAATTTTGGATCCATCGACGGGGA 240

L2b/UCH-1/proctitis TTGGGCCATGCGATACCCTCTTGTTGATGGTCAAGGGAATTTTGGATCCATCGACGGGGA 240

E/11023 TTGGGCCATGCGATACCCTCTTGTTGATGGTCAAGGGAATTTTGGATCCATCGACGGGGA 240

Sweden2 TTGGGCCATGCGATACCCTCTTGTTGATGGTCAAGGGAATTTTGGATCCATCGACGGGGA 240

E/150 TTGGGCCATGCGATACCCTCTTGTTGATGGTCAAGGGAATTTTGGATCCATCGACGGGGA 240

************************************************************

Clinical TCCAGCTGCTGCCATGCGATATACAGAGGCTCGCCTGACTCACAGCGCTATCTTTTTGTT 300

D-LC TCCAGCTGCTGCCATGCGATATACAGAGGCTCGCCTGACTCACAGCGCTATCTTTTTGTT 300

D-EC TCCAGCTGCTGCCATGCGATATACAGAGGCTCGCCTGACTCACAGCGCTATCTTTTTGTT 300

G/9301 TCCAGCTGCTGCCATGCGATATACAGAGGCTCGCCTGACTCACAGCGCTATCTTTTTGTT 300

G/11074 TCCAGCTGCTGCCATGCGATATACAGAGGCTCGCCTGACTCACAGCGCTATCTTTTTGTT 300

A/HAR-13 TCCAGCTGCTGCCATGCGATATACAGAGGCTCGCCTGACTCACAGCGCTATCTTTTTGTT 300

D/UW-3/CX TCCAGCTGCTGCCATGCGATATACAGAGGCTCGCCTGACTCACAGCGCTATCTTTTTGTT 300

B/Jali20/OT TCCAGCTGCTGCCATGCGATATACAGAGGCTCGCCTGACTCACAGCGCTATCTTTTTGTT 300

B/TZ1A828/OT TCCAGCTGCTGCCATGCGATATACAGAGGCTCGCCTGACTCACAGCGCTATCTTTTTGTT 300

L2b/UCH-1/proctitis TCCAGCTGCTGCCATGCGATATACAGAGGCTCGCCTGACTCACAGCGCTATCTTTTTGTT 300

E/11023 TCCAGCTGCTGCCATGCGATATACAGAGGCTCGCCTGACTCACAGCGCTATCTTTTTGTT 300

Sweden2 TCCAGCTGCTGCCATGCGATATACAGAGGCTCGCCTGACTCACAGCGCTATCTTTTTGTT 300

E/150 TCCAGCTGCTGCCATGCGATATACAGAGGCTCGCCTGACTCACAGCGCTATCTTTTTGTT 300

************************************************************

Clinical AGAGGACCTAGATAAAGATACTGTAGATATGGTCCCTAACTACGATGAAACTAAATATGA 360

D-LC AGAGGACCTAGATAAAGATACTGTAGATATGGTCCCTAACTACGATGAAACTAAATATGA 360

D-EC AGAGGACCTAGATAAAGATACTGTAGATATGGTCCCTAACTACGATGAAACTAAATATGA 360

G/9301 AGAGGACCTAGATAAAGATACTGTAGATATGGTCCCTAACTACGATGAAACTAAATATGA 360

G/11074 AGAGGACCTAGATAAAGATACTGTAGATATGGTCCCTAACTACGATGAAACTAAATATGA 360

A/HAR-13 AGAGGACCTAGATAAAGATACTGTAGATATGGTCCCTAACTACGATGAAACTAAATATGA 360

D/UW-3/CX AGAGGACCTAGATAAAGATACTGTAGATATGGTCCCTAACTACGATGAAACTAAATATGA 360

B/Jali20/OT AGAGGACCTAGATAAAGATACTGTAGATATGGTCCCTAACTACGATGAAACTAAATATGA 360

B/TZ1A828/OT AGAGGACCTAGATAAAGATACTGTAGATATGGTCCCTAACTACGATGAAACTAAATATGA 360

L2b/UCH-1/proctitis AGAGGACCTAGATAAAGATACTGTAGATATGGTCCCTAACTACGATGAAACTAAATATGA 360

E/11023 AGAGGACCTAGATAAAGATACTGTAGATATGGTCCCTAACTACGATGAAACTAAATATGA 360

Sweden2 AGAGGACCTAGATAAAGATACTGTAGATATGGTCCCTAACTACGATGAAACTAAATATGA 360

E/150 AGAGGACCTAGATAAAGATACTGTAGATATGGTCCCTAACTACGATGAAACTAAATATGA 360

************************************************************

Clinical ACCTGTAGTTTTTCCTTCAAAATTCCCCAATTTACTTTGTAATGGCTCCTCAGGCATCGC 420

D-LC ACCTGTAGTTTTTCCTTCAAAATTCCCCAATTTACTTTGTAATGGCTCCTCAGGCATCGC 420

D-EC ACCTGTAGTTTTTCCTTCAAAATTCCCCAATTTACTTTGTAATGGCTCCTCAGGCATCGC 420

G/9301 ACCTGTAGTTTTTCCTTCAAAATTCCCCAATTTACTTTGTAATGGCTCCTCAGGCATCGC 420

G/11074 ACCTGTAGTTTTTCCTTCAAAATTCCCCAATTTACTTTGTAATGGCTCCTCAGGCATCGC 420

A/HAR-13 ACCTGTAGTTTTTCCTTCAAAATTCCCCAATTTACTTTGTAATGGCTCCTCAGGCATCGC 420

D/UW-3/CX ACCTGTAGTTTTTCCTTCAAAATTCCCCAATTTACTTTGTAATGGCTCCTCAGGCATCGC 420

B/Jali20/OT ACCTGTAGTTTTTCCTTCAAAATTCCCCAATTTACTTTGTAATGGCTCCTCAGGCATCGC 420

B/TZ1A828/OT ACCTGTAGTTTTTCCTTCAAAATTCCCCAATTTACTTTGTAATGGCTCCTCAGGCATCGC 420

L2b/UCH-1/proctitis ACCTGTAGTTTTTCCTTCAAAATTCCCCAATTTACTTTGTAATGGCTCCTCAGGCATCGC 420

E/11023 ACCTGTAGTTTTTCCTTCAAAATTCCCCAATTTACTTTGTAATGGCTCCTCAGGCATCGC 420

Sweden2 ACCTGTAGTTTTTCCTTCAAAATTCCCCAATTTACTTTGTAATGGCTCCTCAGGCATCGC 420

E/150 ACCTGTAGTTTTTCCTTCAAAATTCCCCAATTTACTTTGTAATGGCTCCTCAGGCATCGC 420

************************************************************

Clinical GGTAGGGATGGCAACAAATATTCCACCGCATAATTTAGGGGAA 463

D-LC GGTAGGGATGGCAACAAATATTCCACCGCATAATTTAGGGGAA 463

D-EC GGTAGGGATGGCAACAAATATTCCACCGCATAATTTAGGGGAA 463

G/9301 GGTAGGGATGGCAACAAATATTCCACCGCATAATTTAGGGGAA 463

G/11074 GGTAGGGATGGCAACAAATATTCCACCGCATAATTTAGGGGAA 463

A/HAR-13 GGTAGGGATGGCAACAAATATTCCACCGCATAATTTAGGGGAA 463

D/UW-3/CX GGTAGGGATGGCAACAAATATTCCACCGCATAATTTAGGGGAA 463

B/Jali20/OT GGTAGGGATGGCAACAAATATTCCACCGCATAATTTAGGGGAA 463

B/TZ1A828/OT GGTAGGGATGGCAACAAATATTCCACCGCATAATTTAGGGGAA 463

L2b/UCH-1/proctitis GGTAGGGATGGCAACAAATATTCCACCGCATAATTTAGGGGAA 463

E/11023 GGTAGGGATGGCAACAAATATTCCACCGCATAATTTAGGGGAA 463

Sweden2 GGTAGGGATGGCAACAAATATTCCACCGCATAATTTAGGGGAA 463

E/150 GGTAGGGATGGCAACAAATATTCCACCGCATAATTTAGGGGAA 463

***************************************************
